# Conditionally reprogrammed primary airway epithelial cells maintain morphology, lineage and disease specific functional characteristics

**DOI:** 10.1038/s41598-017-17952-4

**Published:** 2017-12-21

**Authors:** Kelly M. Martinovich, Thomas Iosifidis, Alysia G. Buckley, Kevin Looi, Kak-Ming Ling, Erika N. Sutanto, Elizabeth Kicic-Starcevich, Luke W. Garratt, Nicole C. Shaw, Samuel Montgomery, Francis J. Lannigan, Darryl A. Knight, Anthony Kicic, Stephen M. Stick

**Affiliations:** 1 Telethon Kids Institute, Centre for Health Research, The University of Western Australia, Crawley, Western Australia Australia; 2 School of Paediatrics and Child Health, The University of Western Australia, Crawley, Western Australia Australia; 3 Centre for Cell Therapy and Regenerative Medicine, School of Medicine and Pharmacology, The University of Western Australia, Nedlands, Western Australia Australia; 4 Centre of Microscopy, Characterisation and Analysis, The University of Western Australia, Crawley, Western Australia Australia; 5 School of Biomedical Sciences and Pharmacy, University of Newcastle, Callaghan, New South Wales Australia; 6 Priority Research Centre for Asthma and Respiratory Disease, Hunter Medical Research Institute, Newcastle, New South Wales Australia; 7 Department of Anesthesiology, Pharmacology and Therapeutics, University of British Columbia, Vancouver, Canada; 8 Department of Respiratory Medicine, Princess Margaret Hospital for Children, Perth, Western Australia Australia; 9 Occupation and Environment, School of Public Health, Curtin University, Perth, Western Australia Australia

## Abstract

Current limitations to primary cell expansion led us to test whether airway epithelial cells derived from healthy children and those with asthma and cystic fibrosis (CF), co-cultured with an irradiated fibroblast feeder cell in F-medium containing 10 µM ROCK inhibitor could maintain their lineage during expansion and whether this is influenced by underlying disease status. Here, we show that conditionally reprogrammed airway epithelial cells (CRAECs) can be established from both healthy and diseased phenotypes. CRAECs can be expanded, cryopreserved and maintain phenotypes over at least 5 passages. Population doublings of CRAEC cultures were significantly greater than standard cultures, but maintained their lineage characteristics. CRAECs from all phenotypes were also capable of fully differentiating at air-liquid interface (ALI) and maintained disease specific characteristics including; defective CFTR channel function cultures and the inability to repair wounds. Our findings indicate that CRAECs derived from children maintain lineage, phenotypic and importantly disease-specific functional characteristics over a specified passage range.

## Introduction

The study of the respiratory epithelium is critical to many chronic lung diseases such as cystic fibrosis (CF) and asthma. Work by us and others, suggests a dynamic and critical role of primary airway epithelial cells (pAEC) in the pathogenesis of chronic lung diseases^1–5^. Until recently, the difficulty in obtaining target organ tissue from patients, specifically in children, has meant that most information regarding these diseases has been derived from studies performed in immortalised cell lines, animal models or tissue from adults^6–8^. We and others have successfully adapted, implemented and optimised a method to obtain airway epithelial cells (AEC) by airway brushing in children^1,9–11^ providing a primary cell source which subsequently has been used to establish cultures for the study of paediatric lung diseases.

There are, however several limitations in primary AEC culture establishment. Firstly, cell yields and viability from airway brushings are highly variable. Secondly, primary cell cultures take 10–14 days to fully establish before being expanded via serial passage^1^. Finally, primary cells have a very limited proliferative capacity *in vitro*, undergoing approximately 3–4 population doublings before becoming senescent^1^. Ideally, cultures should be successfully established from every brushing irrespective of disease phenotype, have greater proliferative capacity to enable expansion beyond current limits and allow for multiple downstream experiments using matched samples. Critically, these cultures should maintain their epithelial lineage and phenotypic characteristics. Here, we describe such a methodology utilising defined additives and feeder cell layer.

The concept of co-culturing multiple cell types together to improve proliferation is relative commonplace^12,13^. Fibroblast-epithelial co-culture models include those from the nose^14,15^, cornea^16^, particular tumours^17,18^ and skin keratinocytes^19^. However, this cell co-culture method has not yet been applied for hard to obtain tissues, including pAECs from children. Cell co-culture systems commonly utilise the murine embryonic fibroblast cell line, NIH-3T3 as an irradiated feeder cell layer as well as a Rho-associated kinase (ROCK) inhibitor that has been shown to increase population doublings and proliferation of prostate and breast epithelial cells while retaining original karyotypes^20–22^. We have chosen here to use these two conditions to assess their effect on primary paediatric AEC cultures which we term conditionally reprogrammed airway epithelial cells (CRAECs). We were particularly interested to determine whether these culture conditions preserved the phenotypic and functional abnormalities seen in cells from children with asthma or CF.

In this study, we tested the hypothesis that cell morphology, epithelial mRNA and protein expression profiles as well as functional changes of AEC grown under co-culture conditions are maintained while the population doubling capacity and cell yield is significantly improved compared to our standard culture method. Results generated in this study, show that co-culturing AECs with irradiated NIH-3T3 fibroblast cells and in the presence of a ROCK inhibitor significantly improves pAEC growth rates and extends the number of population doublings *in vitro*. This methodology significantly increases the total number of cells available for experimental purposes whilst retaining epithelial lineage characteristics and disease specific abnormal functionalities over a specified passage range.

## Results

### Cell Culture improvements

**Figure 1:**
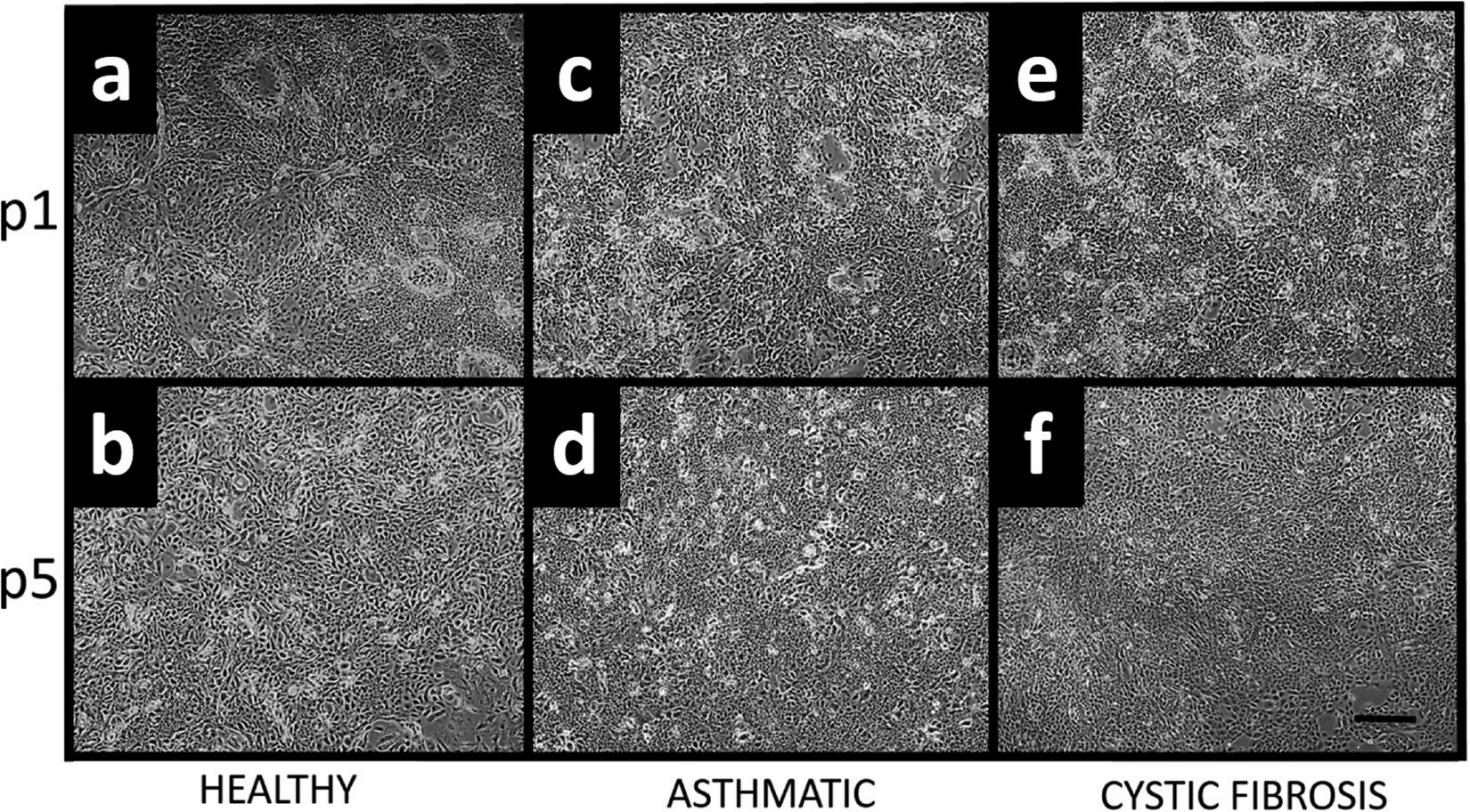
Epithelial cobblestone morphology is maintained over passage. **(a)** Healthy CRAEC monolayer at confluence, passage one. (**b)** Healthy CRAEC monolayer at confluence, passage five. (**c)** Asthmatic CRAEC monolayer at confluence, passage one. (**d)** Asthmatic CRAEC monolayer at confluence, passage five. (**e)** Cystic fibrosis CRAEC monolayer at confluence, passage one. (**f)** Cystic fibrosis CRAEC monolayer at confluence, passage five. Representative image of n = 4 patients per phenotype/passage. Scale bar: 100 µm.

**Figure 2:**
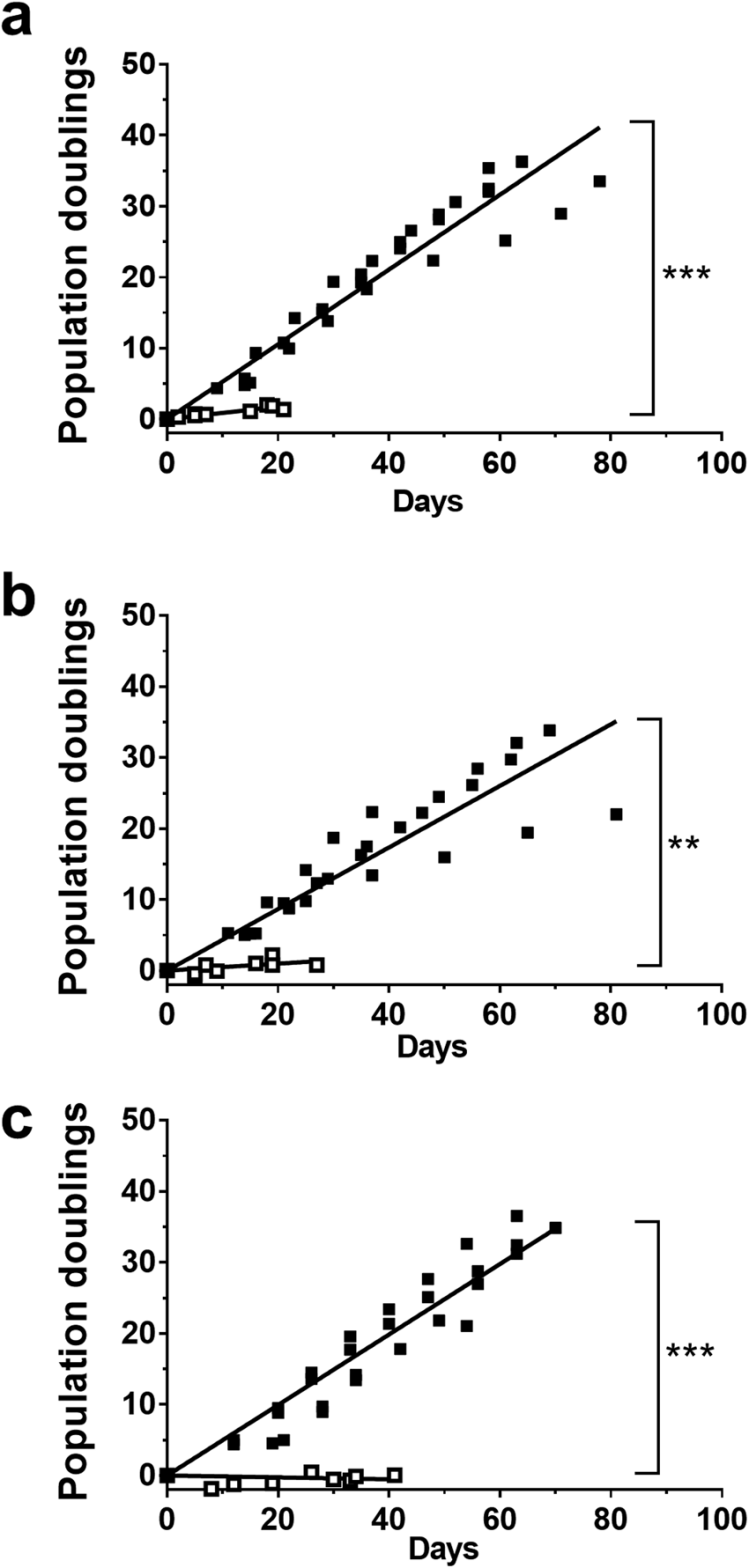
Conditionally reprogramming improves cell population doubling potential. **(a)** Population doublings of healthy AECs (n = 4 patients; CRAEC slope 0.52 ± 0.02; pAEC slope 0.08 ± 0.01; p = 0.0001). (**b)** Population doublings of asthmatic AECs (n = 4 patients; CRAEC slope 0.40 ± 0.03; pAEC slope 0.06 ± 0.02; p = 0.0050). (**c)** Population doublings of CF AEC (n = 4 patients; CRAEC slope 0.50 ± 0.01; pAEC slope 0.06 ± 0.02; p = 0.0001). CRAEC ■, pAEC □, ***p < 0.0010, **p < 0.0100.

CRAECs exhibited a characteristic epithelial cobblestone morphology which was maintained throughout the life of every culture. There were no observed morphological differences between healthy (Fig. 1a & b), asthmatic (Fig. 1c & d) and CF (Fig. 1e & f) cultures over passages 1 to 5. Conditional reprogramming of pAEC cultures improved the population doubling times of both the healthy and disease phenotypes compared to standard cultures for healthy (CRAEC slope 0.56 ± 0.02, pAEC 0.08 ± 0.01; p = 0.0001; Fig. 2a), asthmatic (CRAEC slope 0.41 ± 0.03, pAEC 0.06 ± 0.02; p = 0.0048 Fig. 2b) and CF cultures (CRAEC slope 0.50 ± 0.01, pAEC 0.06 ± 0.02; p = 0.0001; Fig. 2c). When assessed over time, population doubling rates of cultures at passage 1 were not significantly different between healthy (5.10 ± 0.26) and CF (4.51 ± 0.06) (p = 0.0702), however were between heathy and asthmatic phenotypes (4.23 ± 0.22) (p = 0.0464). No significant difference was seen between asthmatic and CF phenotypes at this passage (p = 0.2856). When doubling rates were assessed at passage 5, no significant differences were observed between any of the phenotypes (healthy 3.64 ± 0.36, Asthmatic 3.90 ± 0.47, CF 4.10 ± 0.406).

**Figure 3:**
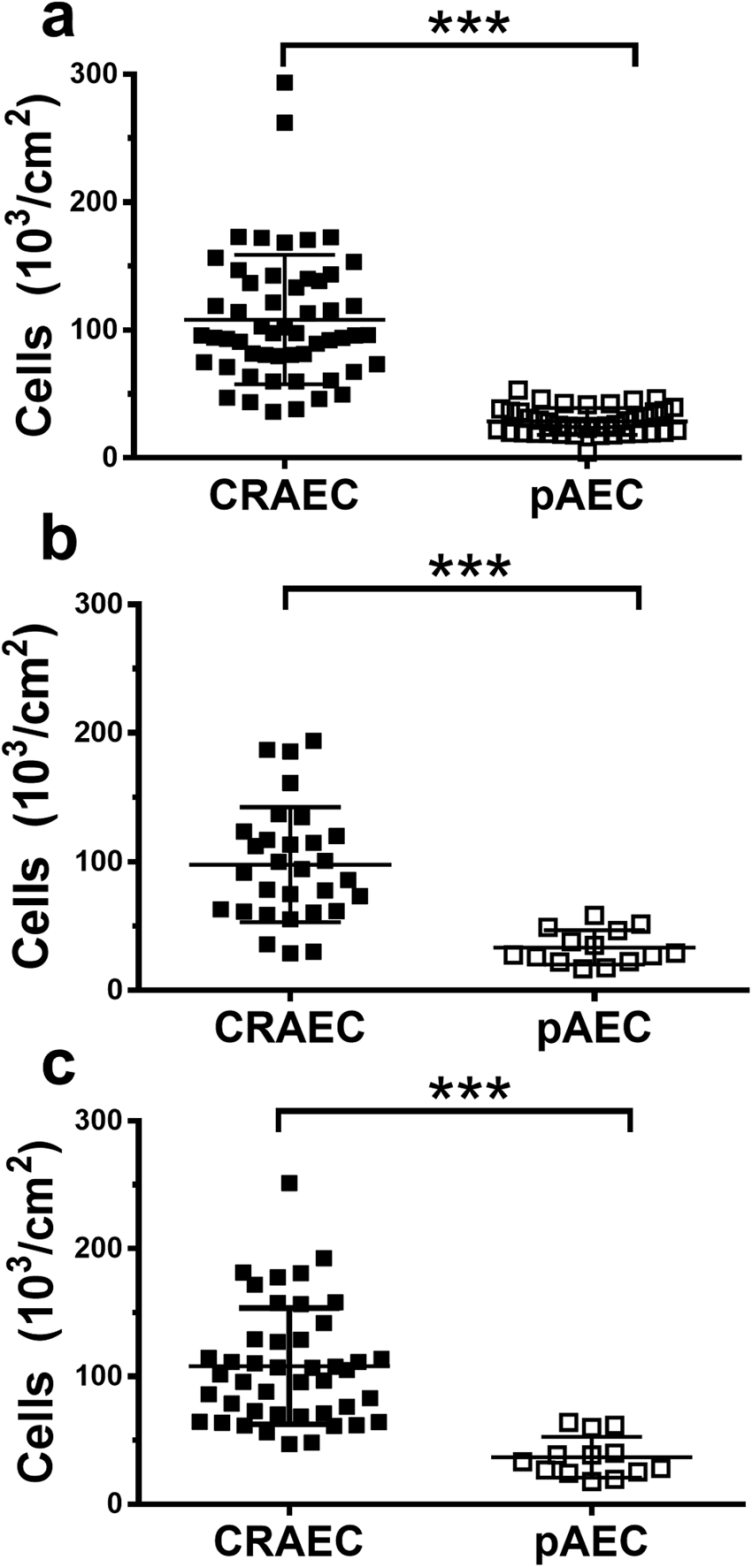
Cell yield is improved by conditional reprogramming. **(a)** Cell yield of healthy AECs (CRAEC 108.2 ± 50.63 thousand cells per cm^2^, pAEC 28.6 ± 10.54 thousand cells per cm^2^, p = 0.0001). (**b**) Cell yield of asthmatic AECs (CRAEC 97.8 ± 44.68 thousand cells per cm^2^ pAEC 33.43 ± 13.36 thousand cells per cm^2^, p = 0.0001) (**c)** Cell yield of CF AECs (CRAEC 108.1 ± 45.4 thousand cells per cm^2^, pAEC 36.86 ± 15.97 thousand cells per cm^2^, p = 0.0001) CRAEC ■, pAEC □, ***p < 0.0010.

Cell yields were also significantly improved with conditional reprogramming; healthy (CRAEC 108.20 ± 50.63 × 10^3^ cells/cm^2^ vs pAEC 28.60 ± 10.54 × 10^3^ cells/cm^2^, p = 0.0001; Fig. 3a), asthmatic (CRAEC 97.80 ± 44.68 × 10^3^ cells/cm^2^ vs pAEC 33.43 ± 13.36 × 10^3^ cells/cm^2^, p = 0.0001; Fig. 3b) and CF (CRAEC 108.10 ± 45.40 × 10^3^ cells/cm^2^ vs pAEC 36.86 ± 15.97 × 10^3^ cells/cm^2^, p = 0.0001 Fig. 3c). The improved cell yield facilitated routine cryopreservation of all cultures and improved their recovery capacity. After at least 1 month in liquid nitrogen storage, CRAECs of various passages were thawed. High cell viabilities were achieved for each phenotype recovered; healthy (75.77% ± 8.62%), asthmatic (88.50% ± 5.07%) and CF (83.79% ± 10.20%).

### Epithelial lineage is maintained

**Figure 4:**
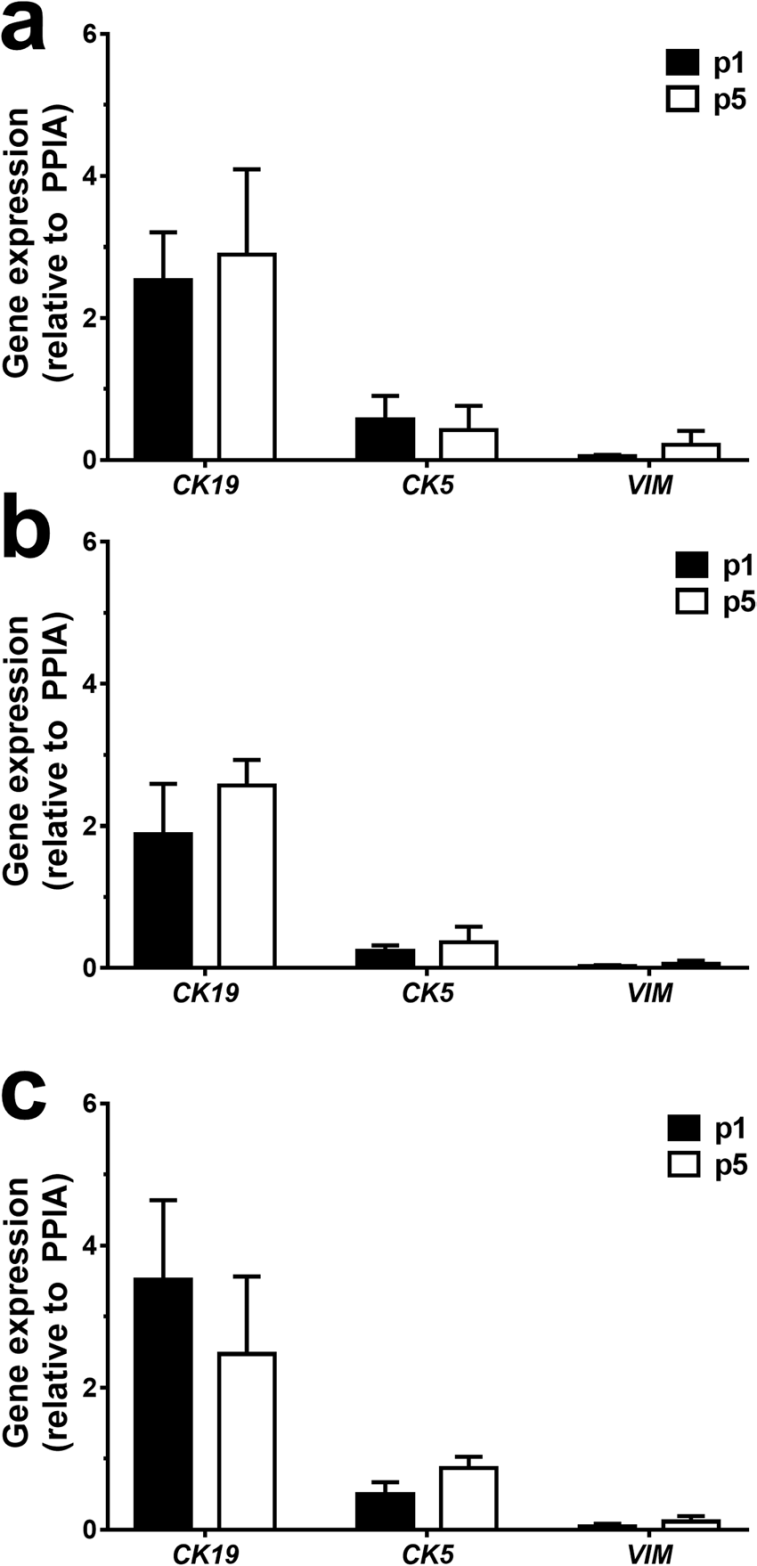
Gene expression of cytokeratin 19, cytokeratin 5 and vimentin is maintained over passage. **(a)** Gene expression profile of healthy CRAECs from passage one and five. (**b)** Gene expression profile of asthmatic CRAECs from passage one and five. (**c)** Gene expression profile of CF CRAECs from passage one and five. Passage 1 (black bar), passage 5 (open bar). No significant differences between passages or phenotypes. (n = 4 patients per phenotype/passage, relative expression to housekeeping gene, *PPIA*).

Maintenance of epithelial lineage over passage was confirmed via two methods; qPCR and immunocytochemistry. No statistical differences were found for *CK19*, *CK5* or *VIM* gene expression between passage one and five for all three phenotypes (Fig. 4). Expression of epithelial gene *CK19* was significantly higher than that the mesenchymal marker *VIM* in all three phenotypes; healthy (p1: *CK19* 2.53 ± 0.67 *VIM* 0.05 ± 0.02 p = 0.01; p5: *CK19* 2.89 ± 1.20 *VIM* 0.22 ± 0.19 p = 0.02; Fig. 4a) asthmatic (p1: *CK19* 1.88 ± 0.71 *VIM* 0.02 ± 0.02 p = 0.01; p5: *CK19* 2.57 ± 0.36 *VIM* 0.06 ± 0.05 p = 0.01; Fig. 4b) and CF (p1: *CK19* 3.52 ± 1.12 *VIM* 0.04 ± 0.04 p = 0.01; p5: *CK19* 2.47 ± 1.09 *VIM* 0.12 ± 0.08 p = 0.01; Fig. 4c). *CK5* expression was also significantly higher than *VIM* expression and maintained over extended passage and between all phenotypic groups (p1: *CK5* 0.57 ± 0.33 *VIM* 0.05 ± 0.02 p = 0.02, p5: *CK5* 0.42 ± 0.34 *VIM* 0.22 ± 0.20 p = 0.02) asthmatic (p1: *CK5* 0.24 ± 0.08 *VIM* 0.02 ± 0.02 p = 0.01, p5: *CK5* 0.36 ± 0.22 *VIM* 0.06 ± 0.05 p = 0.04) and CF (p1: *CK5* 0.50 ± 0.17 *VIM* 0.04 ± 0.04 p = 0.01, p5: *CK5* 0.86 ± 0.16 *VIM* 0.12 ± 0.08 p = 0.01).

**Figure 5:**
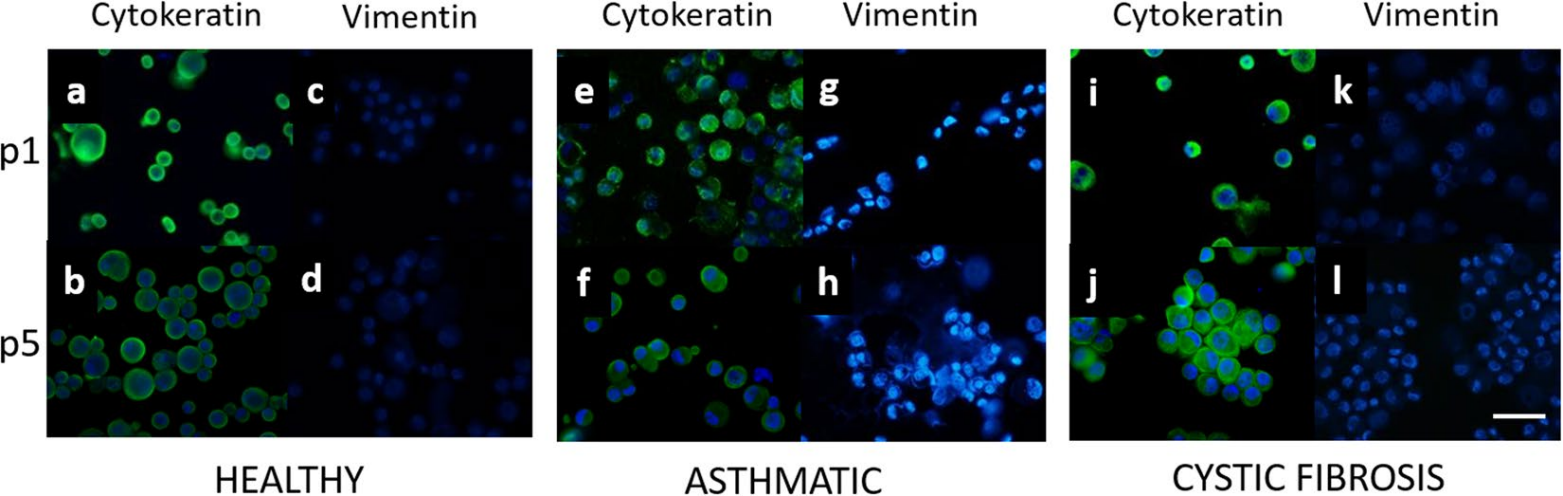
Expression of the epithelial lineage marker cytokeratin is maintained over passage. Pan-cytokeratin antibody (DAKO) **(a**,**b**,**e**,**f**,**i**,**j)**, Vimentin antibody (Abcam), **(c**,**d**,**g**,**h**,**k**,**l)**, DAPI nuclear stain represented in blue on all images. Representative images n = 4 patients per phenotype/passage, Scale bar; 30 µm.

Gene expression was subsequently validated at the protein level using immunocytochemistry. Staining of CRAEC cytospins at both passage 1 and 5 with the sentinel epithelial marker AE1/AE3, displayed very strong positive immunostaining that was maintained over passage. This was replicated and observed in all three phenotypes; healthy (Fig. 5a & b), asthmatic (Fig. 5e & f) and CF (Fig. 5i & j). In contrast, expression of vimentin was minimally detected, if at all, in either passage 1 or 5 in any of the three phenotypes assessed; healthy (Fig. 5c & d) asthmatic (Fig. 5g & h) and CF (Fig. 5k & l).

### Functional differentiation capacity is retained

#### Airway epithelial cell differentiation

**Figure 6:**
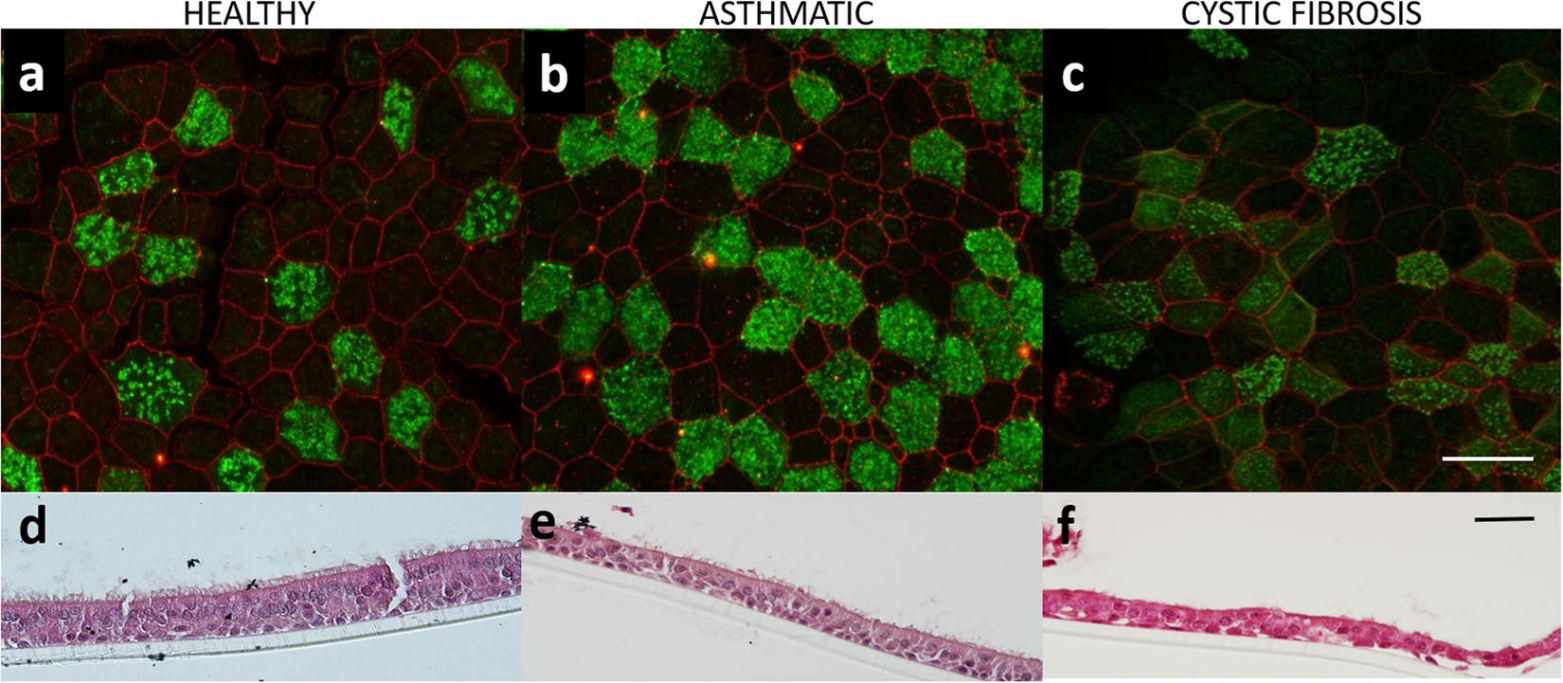
Terminal differentiation at the air-liquid interface (ALI). **(a)** Differentiation expression profile of healthy CRAECs. (**b)** Differentiation expression profile of asthmatic CRAECs. (**c)** Differentiation expression profile of CF CRAECs. Cilia shown in green, stained using α-tubulin, tight junction protein ZO-1 shown in red (oil immersion, Scale bar; 50 µm). (**d)** Cross section of healthy CRAECs. (**e)** Cross section of asthmatic CRAECs. (**f)** Cross section of Cystic fibrosis CRAECs. Sections stained with H&E, apical side is the uppermost. (Scale bar; 60 µm; representative images, n = 4 patients per phenotype/per passage).

All conditionally reprogrammed cultures grown at the ALI stratified into multiple cell layers and differentiated into ciliated and mucus-producing cells (Fig. 6a–f). Mucociliary differentiated CRAEC passage one ALI cultures also stained positively for the typically expressed tight junctional protein, ZO-1 and α-tubulin which stain cilia (Fig. 6a–c). Fixed ALI cultures that were sectioned and stained illustrated multiple cell layers and cilia on terminally differentiated cells located at the apical surface (Fig. 6d–f). Cryopreserved CRAEC cultures which were thawed, expanded and grown at the ALI at passage two and five exhibited similar differentiation capacity (Supplementary Fig. 1a–d).

#### CFTR function

**Figure 7:**
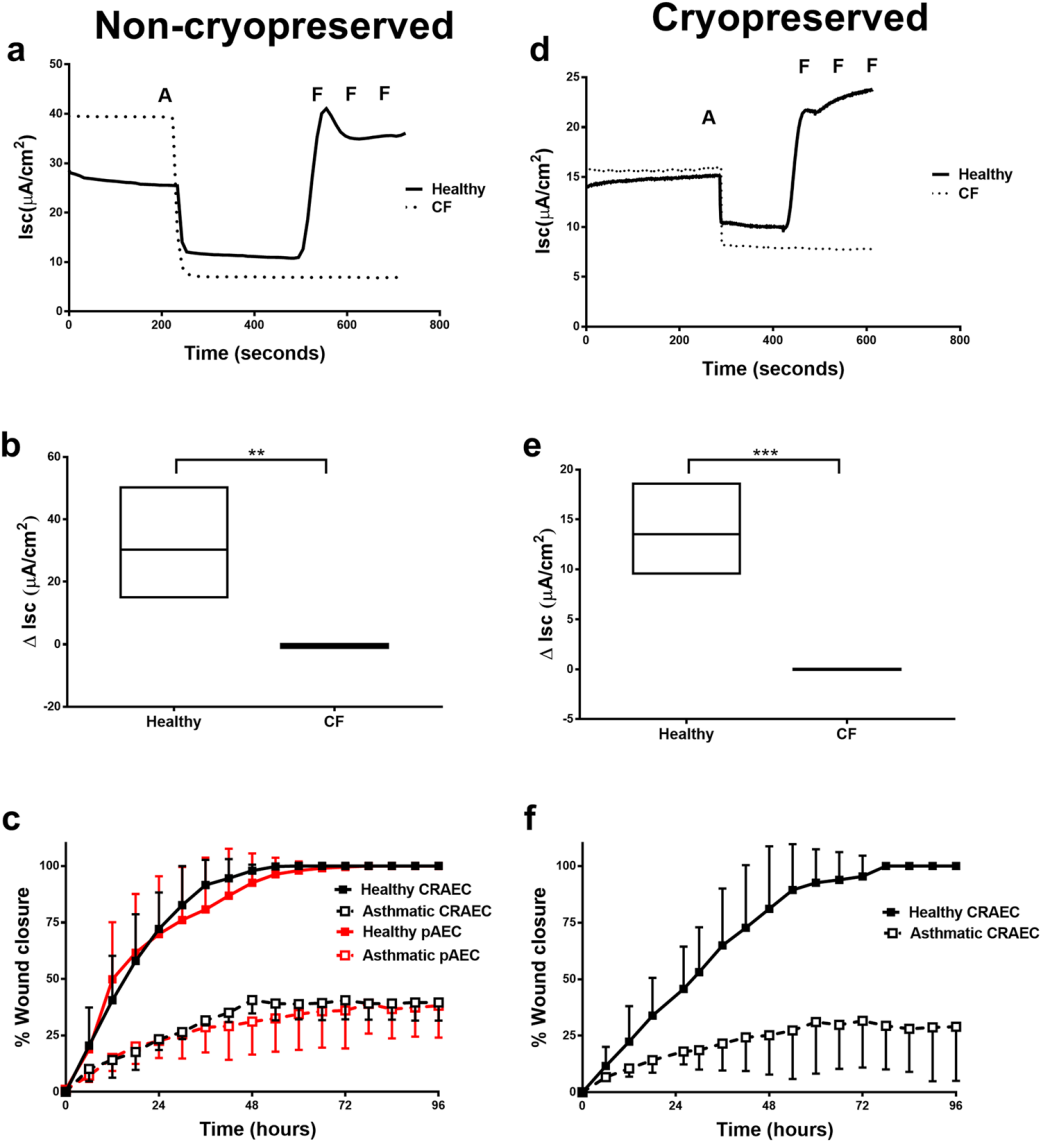
Disease specific functional characteristics are maintained in non-cryopreserved and cryopreserved CRAECs. **(a)** Ussing chamber studies utilising differentiated non-cryopreserved ALI cultures from a healthy phenotype have functional CFTR (solid line) whereas CF cultures do not (dotted line). Amiloride treatment (A) blocks sodium ion adsorption, forskolin treatment (F) stimulates CFTR driven chloride ion secretion. Representative tracings of short circuit current (Isc), n = 4 CF patients, n = 4 healthy patients. (**b)** Change in Isc in Ussing chamber studies, after the addition of forskolin in healthy and CF non-cryopreserved ALI cultures. Floating bars shown of the min and max with line at the mean, n = 4 CF patients, n = 4 healthy patients **p = 0.0060. (**c)** Asthmatic pAECs and CRAECs have a dysregulated wound repair capacity. Mechanical scratch wounds were performed on pAEC (red) and CRAEC (black) submerged monolayer cultures from non-cryopreserved healthy (solid line & solid squares) and asthmatic children (dashed line & open squares). Wound closure was calculated by manual tracing of the new wound area at each time interval, then expressed as a percentage of total wound recovery. Both pAECs and CRAECs from asthmatic children (dashed line & open squares) failed to repair. (n = 4 healthy patients, asthmatic patients, each performed in technical duplicates at passage two). (**d)** Ussing chamber studies utilising differentiated cryopreserved ALI cultures from a healthy phenotype have functional CFTR (solid line) whereas CF cultures do not (dotted line). Amiloride treatment (A) blocks sodium ion adsorption, forskolin treatment (F) stimulates CFTR driven chloride ion secretion. Representative tracings of Isc, n = 4 CF patients and n = 4 healthy patients. (**e)** Change in Isc in Ussing chamber studies, after the addition of forskolin in healthy and CF cryopreserved ALI cultures. Floating bars shown of the min and max with line at the mean, n = 4 CF patients, n = 4 healthy patients ***p = 0.0040. (**f)** Mechanical scratch wounds were performed on CRAEC submerged monolayer cultures from cryopreserved healthy (solid line & solid squares) and asthmatic children (dashed line & open squares) and wound repair monitored. Cryopreserved asthmatic CRAECs failed to repair. (n = 4 healthy patients, asthmatic patients, each performed in technical duplicates at passage two).

Mucociliary differentiated ALI cultures established from children without CF, both non- cryopreserved and cryopreserved cells were observed to retain CFTR function. In the presence of the Na+ channel blocker amiloride (Fig. 7a & d; solid line (A)); forskolin stimulated CFTR driven chloride ion secretion was shown by an increase in short circuit current (*Isc*) (Fig. 7a & d; solid line (F)) and this continued to increase with the repeated addition of forskolin. In contrast, ALI cultures established from children with CF responded to amiloride treatment, as indicated by the drop in *Isc* (Fig. 7a & d; dotted line (A)). However, cultures did not respond to repeated addition of forskolin, indicating a non-functional CFTR (Fig. 7a & d; dotted line (F)) and the retention of dysfunctional CFTR. The combined change in *Isc* for non-cryopreserved healthy CRAECs (30.21 ± 7.36 µA/cm^2^) was significantly greater than CF CRAECs (−0.29 ± 0.26 µA/cm^2^; p = 0.0060) (Fig. 7b). This phenotypic functional difference was maintained in cryopreserved cultures at passage two (Fig. 7e) (Heathy 13.54 ± 1.87 µA/cm^2^; CF 0.01 ± 0.02 µA/cm^2^; p = 0.0010). This phenotypic difference was also maintained after cryopreservation and five passages (Heathy 8.00 ± 0.95 µA/cm^2^; CF 0.06 ± 0.08 µA/cm^2^; p = 0.0010) (Supplementary Fig. 2a & b).

#### Wound repair

We performed monolayer scratch wound repair experiments as we have previously shown this to be defective in pAECs from children with asthma^23,24^. pAEC wound repair is shown in Fig. 7c (Healthy pAECs repaired by 57.00 ± 14.28 hours, asthmatic pAECs at 96 hours post wounding 38.18 ± 14.14% closed) to allow direct comparison to the CRAEC wound repair rates. CRAECs derived from both healthy and children with asthma, in both non-cryopreserved and cryopreserved CRAECs were wounded and repair monitored. Expectedly, CRAECs from healthy children migrated into the wound site and fully repaired (Fig. 7c non-cryopreserved repaired by 45.75 ± 11.32 hours; Fig. 7f cryopreserved repaired by 60.00 ± 12.96 hours; solid line). In contrast, CRAECs from children with asthma were unable to fully repair (Fig. 7c; non-cryopreserved at 96 hours post wound 35.59 ± 8.08% closed, Fig. 7f cryopreserved at 96 hours post wound 28.97 ± 23.92% closed; dashed line). There was no significant difference between the wound repair capacity of pAECs and non-cryopreserved CRAECs (Healthy p = 0.2630, Asthmatic p = 0.8680). There was also no significant difference between non-cryopreserved CRAECs and cryopreserved CRAECs (Healthy p = 0.1480, Asthmatic p = 0.4320). Experiments were also repeated after cryopreservation and extended to passage five with no significant differences in repair capacity compared to passage two cryopreserved CRAECs (Healthy p = 0.8939, Asthmatic p = 0.3588) (Supplementary Fig. 2c).

## Discussion

The study of the airway epithelium has provided important insight regarding the pathobiology of lung diseases such as asthma and CF. However, previous limitations of cell yield and expansion capacity of primary cells in culture have limited the number of experiments that can be performed from a single sample. This study has characterised the lineage and functional capacity of paediatric epithelial cells cultured using a recently developed method^20^ based on co-culture with irradiated NIH-3T3 fibroblast feeders and ROCK inhibitor. Here, conditionally reprogrammed cells maintained a cobblestone morphology over passage independent of phenotype and population doublings and cell yields were significantly improved. Epithelial lineage markers were also maintained over a set passage length regardless of phenotype and both healthy and diseased CRAECs maintained their ability to fully differentiate when grown at ALI. Most importantly, we showed that functional characteristics were maintained following conditional reprogramming specifically, CFTR function was lacking in CRAECs derived from children with CF and defective wound repair was maintained in CRAECs from children with asthma. Collectively, our data demonstrate that conditionally reprogramming of pAEC enables rapid expansion of primary airway epithelial cells without compromising their lineage characteristics and important disease phenotype characteristics. Thus, conditional reprogramming provides a platform, using primary cells that can facilitate the study of respiratory diseases, where traditionally, tissue has been difficult to access. More importantly, the most significant immediate benefit lies with the potential use in personalised therapy particularly in CF. Using CRAECs it is now possible to culture sufficient quantities of cells from individuals that can then be used to test the *in vitro* effects of new therapies as they become available and combinations of currently available interventions.

Several papers have begun to characterise the mechanisms that allow cells to be conditionally reprogrammed and have shown that it is the combination of ROCK inhibitor and an irradiated fibroblast feeder layer that are essential, since individually they appear inefficient at cellular reprogramming^20,21,25–27^. Mechanistic studies in multiple cell types have indicated that reprogramming occurs via modulation of a number of molecular pathways, including the suppression of TGF-β and activation of pERK pathways, thus modulating proliferation, differentiation and apoptosis, however none have determined the changes, if any over passage from the basal phenotype^22,25,28^. Interestingly Mou *et al*. used an alternative methodology, namely SMAD signalling inhibition to extend the life of basal epithelial cells, however observed that capacity to differentiate at the ALI was lost over time^29^. Our study successfully characterised key markers of epithelial lineage maintenance over passage in conditionally reprogrammed primary paediatric airway samples isolated from healthy participants, as well as children with mild asthma and children with CF, which are historically more difficult to culture and less readily available^1,30^.

Our data show that the phenotype of CRAEC cultures are maintained over their length in culture (passage 5 here). Maintenance of epithelial morphology and gene and protein expression profiles indicate that the epithelial cells are maintaining their characteristics over passage and not undergoing epithelial-to-mesenchymal transition (EMT) where cells appear elongated and spindle-like and change lineage marker expression^31,32^. Conditional reprogramming of pAECs also extends the culture longevity. Here, we limited our characterisation to passage 5, where cultures were expanded up to 80 days with significantly higher population doublings compared to pAECs. The speed of any population doubling determined in this study were comparable to conditionally reprogrammed mammary epithelial cells and epithelium obtained from bronchial biopsy or nasal brushings^15,27^. In the current study, we stopped cultures at passage 5 which gave a potential cell yield of 90 × 10^10^ cells from 125,000 *ex vivo* cells. CRAECs have however been grown beyond 20 passages in the initial optimisation of this method (data not shown). Combined, these improvements produce an approximate 540-fold increase in cells available for research purposes after just one passage and one week in culture.

An important and highly relevant aspect of AEC cultures is the ability to differentiate when grown at ALI. This model is of emerging importance in asthma research aimed at establishing the effects of pathogens and virus on the integrity of epithelial barrier that is inherently leaky in asthmatics^33–35^. Furthermore, differentiated epithelial cultures are the most appropriate model that can be used to study the mechanism of human rhinovirus (HRV)-C infection in the airway^36–39^. Here, we have shown the successful establishment and maintenance of healthy and disease AEC grown at ALI. We also provided evidence that all cultures up to passage 5 formed multi-cell layers, expressed typical tight junctional proteins and developed cilia. Beyond this, we observed inconsistencies in the ability for CRAECs to fully differentiate. Even so, there is a still a significant improvement (132-fold) in the ability to establish ALIs after just one passage using CRAECs compared to traditional approaches. Collectively, this methodology provides an invaluable expansion resource of matched ALI samples typically needed in the development of new therapies and interventions that have not previously been possible.

We utilised well differentiated ALI cultures for Ussing chamber studies to show that the functional capacity of the ion channels were maintained in non-CF CREACs and defective in CRAECs from children with CF^40^. Depending on their mutation, individuals with CF have differing levels of CFTR function and will respond differently to potential therapeutics^41,42^. Hence, a large culture capacity would be essential when developing and screening novel therapeutic interventions or when trialling drug combinations that have the greatest effect on improving CFTR function. This is a great advantage over using oncogene-transformed cell lines^43^ and end stage (autopsy or explanted lung)^44,45^ samples as the results can be applied clinically to a particular patient that the epithelial cells were obtained from a few weeks prior. Conditional reprogramming also generates a significantly larger number of cells that are mutation and disease specific to an individual that can be cryopreserved. This resource has significant future potential in clinically relevant personalised medicine strategies and airway mucosal research programs.

The major advantage of the current study and its assessment of CRAECs has been its potential use in research programs where primary cell expansion remains a limiting factor. In one such application, we show that CRAECs from healthy children retain the ability to close wounds, whereas CRAECs from children with asthma retain a defective repair capacity similar to their standard culture counterparts^1,23,24^. With our reported findings, this methodology can now be used to accelerate current efforts to elucidate the mechanism of defective wound repair and screen compounds that may restore the reparative capacity in asthmatic patients^46^. As seen with establishing ALI cultures beyond passage 5, we also observed high variability in the reparative capacity of CRAECs within phenotypes at extended passage. With the variability observed particularly at extended passage, comprehensive validation of any functional analysis that utilises conditional reprogramming is required and that results generated without this interpreted with potential caution.

The use of CRAECs for potential cellular based therapies may be somewhat more limiting at present, since there would be the need to eliminate all animal products from the current methodology to comply with regulatory approval. We are currently exploring both substituting the conventional mouse NIH-3T3 that was used in this study^27,47–50^ with a human-derived fibroblast feeder cell layer or removal of the feeder layer entirely. Alternative strategies have utilized the feeder layer to generate a conditioned media, bypassing the need for a co-culture system. The resulting streamlined methodology was seen to improve population doubling rates compared to non-conditionally reprogrammed cells^15^. Additional considerations including FCS, ROCK inhibitor and cholera toxin that are also present in the F-medium would also need to be substituted or titrated out, as successfully performed by LaRanger and colleagues to recellularize mouse lungs^51^. Furthermore, karyotype and tumourigenicity testing would be required prior to the translation of this technology to therapeutic practice.

In summary, we have described how CRAECs derived from healthy children, as well as those with asthma and CF, maintain epithelial lineage marker expression and disease specific functional characteristics. This new method allows for fast establishment, expansion and improved longevity of patient specific airway epithelial cell cultures. The versatile conditional reprogramming methodology can be used to better understand disease pathobiology, for high throughput screening in drug discovery pipelines, and for personalised medicine applications.

## Materials and Methods

### Subjects and primary cell processing

The study was approved by the Princess Margaret Hospital for Children and St John of God Hospital Human Ethics Committees and written consent was obtained from each participant’s legal guardian after being fully informed about the nature and purpose of the study. All experiments were performed in accordance with the relevant committees’ guidelines and regulations. Here, bronchial brushings were obtained from 18 healthy children and 11 children with mild asthma after being admitted into hospital for elective non-respiratory related surgery (Table 1). Children with an existing bacterial or viral chest infection were excluded. Asthma was defined as physician-diagnosed based upon physician documented wheezing episodes in the 12-months preceding their recruitment and confirmed by positive responses on the International Study of Asthma and Allergies in Children (ISAAC) and American Thoracic Society (ATS) respiratory questionnaires^52,53^. All children with asthma had mild intermittent disease and had not had received any asthma medications in the preceding month. Airway brushings were performed as previously described^1,10^ and ~2.67 × 10^6^ AEC were obtained from each child^10,54^. Approximately 5,000 *ex vivo* epithelial cells per cm^2^ were then seeded into a pre-coated and irradiated fibroblast seeded flask (as described below) to establish a CRAEC culture and deemed as passage 0. The remainder of *ex vivo* cells were used for cytospins, RNA, protein and/or establishing a traditional BEGM primary cell culture as previously described^1^. Children with CF (n = 8) had samples collected via bronchoscopy as a component of their annual clinical surveillance program as previously described^55^. Cystic fibrosis transmembrane conductance regulator (CFTR) genotype was determined as part of newborn screening. On average, total cell yields were ~1.4 × 10^6^ per child with CF^30^. Here, 5,000 cells per cm^2^ were used to establish a CRAEC culture as described above, with the remainder used for cytospins, RNA, protein and/or establishing a traditional primary cell culture^55^.

**Table 1:** Patient demographics.

	Healthy	Asthmatic	Cystic Fibrosis
Number	18	11	8
Age (years) ± SD	4.7 ± 3.6	4.1 ± 0.9	3.9 ± 2.1
Sex (Male %)	13/18 (72.2)	6/11 (54.5)	4/8 (50)
Hay fever (%)	4/18 (22.2)	4/11 (36.4)	n/a
Eczema (%)	5/18 (27.8)	5/11 (45.5)	n/a
Phe508del Homozygous (%)	n/a	n/a	4/8 (50)

### Cell types and medium

#### NIH-3T3 murine embryonic fibroblasts

NIH-3T3 murine embryonic fibroblasts were purchased from the American Type Culture Collection (ATCC) (VA, USA) and maintained in Dulbecco’s Modified Eagle Medium (DMEM) growth media (GIBCO, ThermoFisher Scientific Australia) supplemented with 10% (v/v) foetal calf serum (FCS) and 1% (v/v) penicillin/streptomycin (Life Technologies Australia). All cultures were grown at 37 °C in an atmosphere of 5% CO_2_/95% air under aseptic conditions.

#### Irradiating fibroblasts

NIH-3T3 fibroblasts were γ-irradiated prior to use in establishing a CRAEC culture. Fibroblasts were initially trypsinised and irradiated with 3000 cGy γ-radiation (Gammacell® 3000 Elan; MDS Nordion). After γ-irradiation, a total cell count was performed and cells were seeded into a pre-coated tissue culture flask at a density of 5,000 cells per cm^2^ (Corning International) as described^20^.

#### F-medium containing ROCK inhibitor

A specialised F-medium was adapted from Liu and colleagues^20^ for this study. This consisted of; 3:1 (v/v) F-12 Nutrient Mixture (Ham) DMEM (Life Technologies Australia), 5% (v/v) FCS (Life Technologies Australia), 0.4 µg/mL hydrocortisone, 5 µg/mL insulin, 8.4 ng/mL cholera toxin, 10 ng/mL epidermal growth factor, and 24 µg/mL adenine (all Sigma-Aldrich). Finally, 10 µmol/L Y-27632 (ROCK inhibitor) (Enzo Life Sciences) was added to complete the medium.

#### Bronchial Epithelial Growth Medium

Standard primary AEC (pAEC) cultures were also established and expanded in Bronchial Epithelial Basal Medium (BEBM®; LONZA™) supplemented with growth additives and 2% (v/v) Ultroser G (Pall Corporation) as previously described^1,11,23,24^. All cultures were grown at 37 °C in an atmosphere of 5% CO_2_/95% air under aseptic conditions.

### Primary cell subculture

#### Passaging of primary CRAEC

All cultures were grown on tissue culture-treated plastic flasks pre-coated with extracellular matrix components, fibronectin and type I collagen as described^1,11,23,24^. When CRAEC cultures reached approximately 90% confluence, they were passaged by differential trypsinisation using a Trypsin/EDTA reagent pack (LONZA™). This was performed in order to remove feeder cells from the epithelial culture based on their differential trypsin sensitivity as previously described^20^. Briefly, growth medium was aspirated and cells rinsed with a volume of PBS prior to incubation in Tryspin/EDTA at room temperature for 1–2 minutes until fibroblasts had rounded up and lifted off. Cells were rinsed with HEPES-Buffered Saline Solution (HBSS), and incubated in an equal volume of Trypsin/EDTA solution at 37 °C for 5–7 minutes or until epithelial cells had begun to detach from the tissue culture vessel. Cells were then collected, centrifuged at 500 g for 7 minutes at 4 °C, resuspended in F-medium and counted. Viability was also assessed using the trypan-blue exclusion method. The appropriate number of CRAEC were then seeded into pre-coated culture vessels^1^ preseeded with γ-irradiated NIH-3T3 (as described above) to achieve a 1:1 cell ratio and returned to 37 °C in an atmosphere of 5% CO_2_/95% air. Population doubling (PD) was calculated as; PD = 3.32 (log [number of cells harvested/number of cells seeded])^56^. Population doublings were calculated at each passage and shown as accumulative population doublings over days of culture with the line of best fit ± SE indicated. pAEC and CRAEC cultures were then statistically compared using linear regression analysis. Cell yield (CY = cells/cm^2^) was calculated as number of cells harvested x [1 + ((100-% confluence)/100)]/ flask surface area cm^2^.

#### Cryopreservation and thawing assessment

CRAECs were cryopreserved after the initial passage in 1 mL of a cryopreservation solution containing; 10% (v/v) DMSO (Sigma-Aldrich), 90% (v/v) FCS and 10 µmol/L ROCK inhibitor. For thawing assessment, cryopreserved cells were recovered by quick thawing in a 37 °C water bath followed by placement into 9 mL of DMEM containing 10% (v/v) FCS (Life Technologies Australia). Cells were then centrifuged at 500 g for 7 minutes at 4 °C and resuspended in 1 mL of F-medium after which a total cell count and viability were performed. Cells were then seeded into a pre-coated and irradiated NIH-3T3 seeded culture flask at a density of 5,000 cells per cm^2^ in F medium.

### Lineage verification

#### RNA extraction, cDNA synthesis and qPCR

At each passage, 1 million CRAECs were collected, pelleted and resuspended in 350 µL RLT buffer containing 1% (v/v) ß-mercaptoethanol (QIAGEN) and frozen at −80 °C for downstream analysis. RNA was extracted using the Ambion Purelink® RNA mini kit (Thermo Scientific) per manufacturer’s instructions. Genomic DNA was eliminated by on-column RNase-free DNase I digestion (QIAGEN) during isolation. RNA purity and quantity was assessed using the Nanodrop 2000 (Thermo Scientific). Complementary DNA synthesis was carried out as reported previously^57^ and samples stored at −80 °C. Real-time PCR using a SYBR Green protocol^1^ was used to determine the relative gene expression of cytokeratin 5 (*CK5*), cytokeratin 19 (*CK19*), vimentin (*VIM*) and peptidyl-prolyl cis-trans isomerase A (*PPIA*), as outlined in Table 2 ^57^. Relative gene expression was calculated using the 2^−ΔΔCT^ method by normalisation to *PPIA* housekeeping gene and an endogenous tissue control. *PPIA* was chosen as the housekeeping gene of choice due to its uniformity of expression in human epithelial cells derived from healthy and relevant airway disease phenotypes^57^.

**Table 2:** Oligonucleotide Primers.

Gene	Primer	Sequence	Product Length (bp)
Cytokeratin 5 (*CK5*)	Forward	3′-TGGAGATCGCCACTTACCG-5′	109
	Reverse	5′-CCAGAGGAAACACTGCTTGTG-3′	
Cytokeratin 19 (*CK19*)	Forward	3′-CAGCTTCTGAGACCAGGGTT-5′	70
	Reverse	5′-GACTGGCGATAGCTGTAGGA-3′	
Vimentin (*VIM)*	Forward	3′-GAGGAGATGCGGGAGCTG-5′	95
	Reverse	5′-ATGATGTCCTCGGCCAGGTT-3′	
Peptidyl-prolyl cis-trans isomerase A (*PPIA*)	Forward	3′-CCTTGGGCCGCGTCTCCTTT-5′	307
	Reverse	5′-CACCACCCTGACACATAAACCCTGG-3′	

#### Fluorescent immunocytochemistry

Epithelial lineage was confirmed using fluorescent immunocytochemistry as previously described^1,58^. Briefly, fixed cytospun cells were initially rehydrated with 1x PBS and incubated in proteinase K (36 µg/mL; 30 min at 37 °C). Slides were then washed and stained with antibodies specific for either epithelial (AE1-AE3 1:250) (DAKO) or mesenchymal cell lineages (Vimentin 1:250) (Abcam), for 24 hours at 4 °C followed by fluorescently-conjugated secondary antibody (1:1000) (anti-rabbit Alexa Fluor 488 conjugate; Sigma-Aldrich). Cells were then washed again and counterstained with the nuclear specific 4′,6-diamidino-2-phenylindole (DAPI) stain (Sigma-Aldrich). Coverslips were then applied using mounting media (DAKO) and slides imaged using Nikon^®^ Eclipse T*i* inverted microscope (Nikon).

### Functional Assays

#### Airway epithelial cell differentiation

CRAECs utilized in optimization experiments were initially seeded on type IV collagen coated Corning^®^ Transwell^®^ 12 mm 0.4 µm pore polycarbonate membrane cell culture inserts (Sigma-Aldrich) at 150,000 cells/insert in complete bronchial air-liquid interface (B-ALI®) growth medium (LONZA™) supplemented with 10 µmol/L ROCK inhibitor. Once confluent, growth medium was removed from both the apical and basolateral compartments and the insert washed in B-ALI differentiation medium to remove all traces of ROCK inhibitor. B-ALI differentiation medium (LONZA™) was then added into the basolateral compartment only and replaced every 48 hours. Cultures were then monitored for the development of cilia and mucus via microscopy (Nikon).

Validation experiments incorporating both cryopreserved and non-cryopreserved CRAECs were performed using an adapted ALI differentiation media as previously described^59–61^. Briefly, media consisted of a 1:1 mixture of 1xLHC medium and Dulbecco’s Modified Eagle’s Media (DMEM; GIBCO) supplemented with bovine pituitary extract (BPE, 10 μg/ml), hydrocortisone (0.21 μM), human epidermal growth factor (hEGF; 0.5 ng/ml), (L)epinephrine (2.7 μM), insulin (0.87 μM), triiodothyronine (0.01 μM), holotransferrin (0.125 μM), penicillin-G sodium (100 U/ml), streptomycin sulfate (100 μg/ml), bovine serum albumin (0.5 mg/ml), trans-retinoic acid (50 nM), phosphorylethanolamine (0.5 μM), ethanolamine (0.5 μM), zinc sulfate (3 μM), iron (II) sulfate (1.5 μM), magnesium chloride hexahydrate (0.6 mM), calcium chloride dihydrate (1 mM), selenium (30 nM), manganese (1 nM), silicone (500 nM), molybdenum (1 nM), vanadium (5 nM), nickel (1 nM) and tin (0.5 nM) (all Sigma-Aldrich)^59^. CRAECs were seeded on type IV collagen coated Corning^®^ Transwell^®^ 12 mm 0.4 µm pore polycarbonate membrane cell culture inserts (Sigma-Aldrich) at 150,000 cells/insert in ALI^59–61^ medium supplemented with 10 µmol/L ROCK inhibitor. Once confluent, medium was removed from both the apical and basolateral compartments and the insert washed in DMEM to remove all traces of ROCK inhibitor. ALI medium was then added into the basolateral compartment only and replaced every 48 hours. Cultures were then monitored for the development of cilia and mucus via microscopy (Nikon).

Once CRAEC ALI cultures achieved differentiation, they were fixed in ice cold 100% methanol for 10 minutes for antibody staining or Carnoy’s fixative for sectioning. Methanol fixation was followed by a PBS wash at room temperature (RT), and stored at 4 °C in fresh 1 x PBS until stained. Cilia were visualised using α-tubulin (1:300; Sigma-Aldrich clone DM1A), tight junction protein ZO-1 (1:100; 2.5 µg/mL; Life Technologies Australia Clone: ZO1-1A12) and nuclei stain Hoechst 33342 (2.5 μg/mL; Sigma-Aldrich). Secondary antibodies used included; AlexaFluor 488 (1:200; 10 µg/mL; Goat anti-Mouse and Goat anti-Rabbit) and AlexaFluor 568 (1:200; 10 µg/mL; Goat anti-Mouse and Goat anti-Rabbit) (Life Technologies Australia). A Nikon A1 inverted confocal microscope, with a Nikon Plan Apo VC 60x NA 1.4 oil immersion objective and NIS-AR Elements software (v4.2.22, Nikon) was used for imaging. Z-stack images with step size of 0.5 µm were collected with a pinhole of 35.8 µm (1.2 A.U. for 488 nm laser), where the top and bottom of the stacks were determined visually.

CRAEC ALI cultures used for sectioning were fixed in Carnoy’s fixative solution (60% ethanol, 30% chloroform and 10% glacial acetic acid) for 24 hours, rinsed in PBS and then stored at 4 °C in 100% ethanol. Inserts were removed from fixative and placed on a foam pad in a standard histology cassette. After overnight dehydration paraffin infiltration was done using a Leica tissue processor. Inserts were embedded upright for vertical cross-sectioning to show cell layers. After processing, specimens placed into embedding station wax bath. 5um sections were cut and allowed to bake overnight at 60 °C after which they were stained with Hematoxylin and Eosin (H&E) using a Leica Autostainer. An Olympus BX53 microscope was used to visualise the sections.

#### CFTR function

Differentiated CRAEC ALI cultures derived from children with and without CF were established at passage 2 and 5 for chloride ion transport studies using Ussing chamber (Physiologic Instruments Inc.). A chloride ion gradient was established by filling the basolateral compartment with Krebs Ringer bicarbonate solution as previously described^62^. Amiloride (Sigma-Aldrich) was added to the mucosal compartment to a final concentration of 50 µM to block sodium absorption followed by the stepwise addition of mucosal forskolin to a concentration of 0.2–20 µM (F6886 Sigma-Aldrich) to stimulate CFTR-mediated chloride ion secretion.

#### Wound repair assay

Passage 2 pAECs, passage 2 and passage 5 CRAECs were seeded into 12 well plates at a density of 200,000 cells per well in BEBM with SingleQuot supplements (LONZA™), minus epidermal growth factor (EGF) based on previous wound repair assays^23,24,63^. Cells were grown to form a 100% confluent monolayer. To assess wound repair kinetics, a single linear wound was created using a plastic P200 pipette tip (0.5 mm wound width) and time lapse images taken using an IncuCyte ZOOM™ System (Essen BioScience). Wound closure was then calculated as previously described^23,24,63^.

#### Statistical Analysis

Experiments were performed in at least duplicate, with a minimum of 4 patients per experimental cohort. Data are presented as means (±SD) where applicable. Linear regression analysis was used to compare lines of best fit for population doublings and non-parametric Wilcoxon match pairs signed rank tests were used to compare statistical significance between passages. P values less than 0.05 (*), 0.01 (**) and 0.001 (***) were reported in this study to indicate the extent of statistical significance.

## Electronic supplementary material

Supplemental Data
