# Supplementary material for: Conditionally reprogrammed primary airway epithelial cells maintain morphology, lineage and disease specific functional characteristics
Source: Sci Rep. 2017 Dec 21;7:17971. doi: 10.1038/s41598-017-17952-4 (PMC5740081; doi:10.1038/s41598-017-17952-4)
Supplement: Supplementary file 1 — Supplemental Data [file 41598_2017_17952_MOESM1_ESM.doc]

**Conditionally reprogrammed primary airway epithelial cells maintain morphology, lineage and disease specific functional characteristics**

Kelly M. Martinovich^1*^, Thomas Iosifidis^2,3*^, Alysia G. Buckley^4^, Kevin Looi^1^, Kak-Ming Ling^1^, Erika N. Sutanto^1^, Elizabeth Kicic-Starcevich^1^, Luke W. Garratt^1^, Nicole C. Shaw^1^, Samuel Montgomery^1^, Francis J. Lannigan^2^, Darryl A. Knight^5,6,7^, Anthony Kicic ^1,2,3,8,9#^ & Stephen M. Stick ^1,2,3,8#^

**
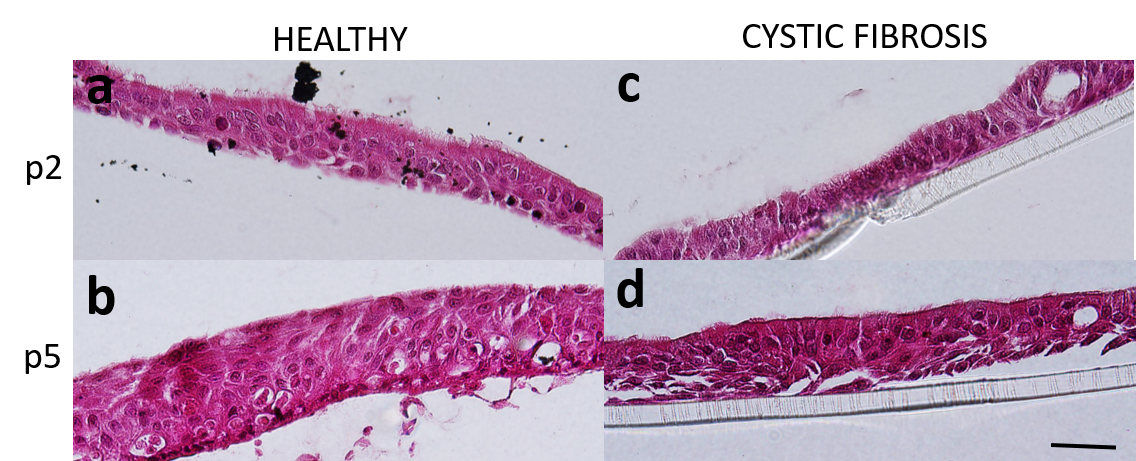
**

**Figure 1. Capacity to terminally differentiate at the air-liquid interface (ALI) is maintained after cryopreservation and extended passage. a)** Cross section of cryopreserved healthy CRAECs grown at the ALI at passage 2. **b)** Cross section of cryopreserved healthy CRAECs grown at the ALI at passage 5. **c)** Cross section of cryopreserved CF CRAECs grown at the ALI at passage 2. **d)** Cross section of cryopreserved CF CRAECs grown at the ALI at passage 5. Sections stained with H & E, apical side is the uppermost. (Scale bar; 60µm; representative images, n=4 patients per phenotype/ per passage).

**
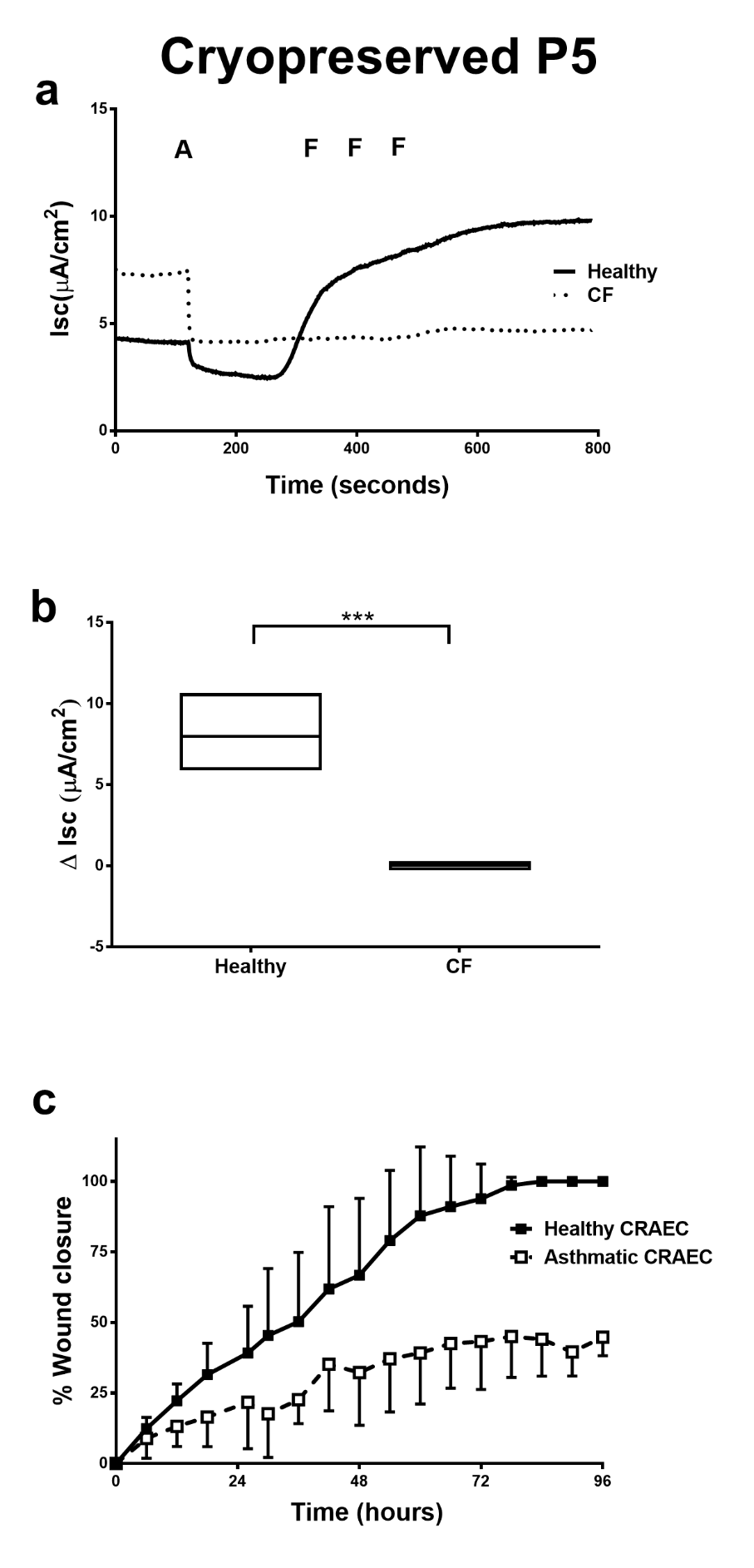
**

**Figure 2. Disease specific functional characteristics are maintained in CRAECs after cryopreservation and extended passage. a)** Ussing chamber studies utilising differentiated cryopreserved ALI cultures from a healthy phenotype have functional CFTR (solid line) whereas CF cultures do not (dotted line). Amiloride treatment (A) blocks sodium ion adsorption, forskolin treatment (F) stimulates CFTR driven chloride ion secretion. Representative tracings of short circuit current (Isc), n=4 CF patients, n=4 healthy patients. **b)** Change in Isc in Ussing chamber studies, after the addition of forskolin in healthy and CF cryopreserved ALI cultures. Floating bars shown of the min and max with line at the mean, n=4 CF patients, n=4 healthy patients ***p=0.0020. **c)** Asthmatic CRAECs maintain their dysregulated wound repair capacity after cryopreservation and extended passage. Mechanical scratch wounds were performed on CRAEC submerged monolayer cultures from cryopreserved healthy (solid line & solid squares) and asthmatic children (dashed line & open squares). Wound closure was calculated by manual tracing of the new wound area at each time interval, then expressed as a percentage of total wound recovery. CRAECs from asthmatic children (dashed line & open squares) failed to repair. (n=4 healthy patients, asthmatic patients, each performed in technical duplicates at passage five).
